# Secretory leukocyte protease inhibitor and risk of heart failure in the Multi-Ethnic Study of Atherosclerosis

**DOI:** 10.1038/s41598-023-27679-0

**Published:** 2023-01-12

**Authors:** Konrad Teodor Sawicki, Drew R. Nannini, Suzette J. Bielinski, Nicholas B. Larson, Donald M. Lloyd-Jones, Bruce Psaty, Kent D. Taylor, Sanjiv J. Shah, Laura J. Rasmussen-Torvik, John T. Wilkins, Elizabeth M. McNally, Ravi B. Patel

**Affiliations:** 1 Division of Cardiology, Department of Medicine, Northwestern University Feinberg School of Medicine, Chicago, IL USA; 2 Center for Genetic Medicine, Northwestern University Feinberg School of Medicine, Chicago, IL USA; 3 Department of Preventive Medicine, Northwestern University Feinberg School of Medicine, Chicago, IL USA; 4 Department of Quantitative Health Sciences, Mayo Clinic, 200 First Street Southwest, Rochester, MN USA; 5 Cardiovascular Health Research Unit, Department of Health Systems and Population Health, University of Washington, Seattle, WA USA; 6 Institute for Translational Genomics, The Lundquist Institute for Biomedical Innovation at Harbor-UCLA Medical Center, Torrance, CA USA; 7 Department of Biochemistry and Molecular Genetics, Northwestern University Feinberg School of Medicine, Chicago, IL USA; 8 Division of Cardiology, Department of Medicine, Northwestern University Feinberg School of Medicine, 676 N St Clair St, Suite 600, Chicago, IL 60611 USA

**Keywords:** Cardiovascular biology, Cardiovascular diseases, Epidemiology, Risk factors, Diagnosis

## Abstract

Circulating protease inhibitors are important regulators of inflammation that are implicated in the pathophysiology of heart failure (HF). Secretory leukocyte protease inhibitor (SLPI) is a serine protease inhibitor which protects pulmonary tissues against inflammatory damage; however, its role in HF is not well understood. We sought to evaluate associations of circulating SLPI and genetically-mediated serum SLPI with incident HF and its subtypes in a multi-ethnic cohort of adults using clinical and genetic epidemiological approaches. Among 2,297 participants in the Multi-Ethnic Study of Atherosclerosis (MESA), each doubling of serum SLPI was independently associated with incident HF (HR 1.77; 95% CI 1.02–3.02; *P* = 0.04), particularly incident HF with preserved ejection fraction (HFpEF; HR 2.44; 95% CI 1.23–4.84; *P* = 0.01) but not HF with reduced ejection fraction (HFrEF; HR 0.95; 95% CI 0.36–2.46; *P* = 0.91). Previously reported circulating SLPI protein quantitative trait loci (pQTLs) were not associated with serum SLPI levels or incident HF among MESA participants. In conclusion, baseline serum SLPI levels, but not genetically-determined serum SLPI, were significantly associated with incident HF and HFpEF over long-term follow-up in a multi-ethnic cohort. Serum circulating SLPI may be a correlate of inflammation that sheds insight on the pathobiology of HFpEF.

## Introduction

Inflammation is a key driver of heart failure (HF) risk^1^. Despite this strong relationship, the mechanisms leading to HF in the setting of inflammation remain unclear. Immune cells secrete a variety of proteolytic enzymes, known as proteases, which can be detected by various methods and contribute to the progression of cardiovascular inflammation^2–7^. HF is characterized by low-grade systemic and cardiac inflammation with immune cell activation, and inhibition of inflammatory mast cell proteases prevents cardiac fibrosis and improves left ventricular dysfunction in animal models^8^. Therefore, the balance between circulating proteases and protease inhibitors may be critical in the modulation of the inflammatory response and pathophysiology of HF.

Secretory leukocyte protease inhibitor (SLPI), also known as antileukoproteinase, is a 11.7 kDa 107-amino acid non-glycosylated protein^9^. SLPI is primarily expressed in human epithelial cells, such as the lining of the respiratory, gastrointestinal, and genitourinary tracts. A second source of SLPI is inflammatory cells, including neutrophils, macrophages, dendritic cells, and mast cells^10^.

Structurally, SLPI consists of two core domains with distinct biological functions; the N-terminal domain provides antimicrobial activity while the C-terminal domain is responsible for protease inhibition^11^. In particular, SLPI inhibits damaging serine proteases, such as neutrophil elastase, chymotrypsin, and cathepsins. Through its anti-proteolytic and antimicrobial properties, SLPI protects local tissues against inflammatory damage and serves as an important regulator of innate and adaptive immunity. Two recent genome-wide association studies (GWAS) of the human blood plasma proteome in European ancestry cohorts identified multiple protein quantitative trait loci (pQTLs) associated with circulating plasma SLPI protein levels^12,13^. However, it is unclear how these SLPI pQTLs contribute to HF risk, particularly in a multi-ethnic population.

The role of serum and genetically-determined SLPI levels in incident HF is unknown. Recent studies demonstrated that elevated serum SLPI was associated with increased risk of incident low ankle-branchial index and atrial fibrillation, suggesting that increased serum SLPI may be predictive of circulatory and other cardiovascular disorders^14,15^. Given the important role of the protease-antiprotease balance in the regulation of inflammation, serum SLPI levels may have important implications for the development of HF, and provide new insights into the mechanisms driving this disease. We thus aimed in this study to evaluate the association of circulating SLPI levels with incident HF and its subtypes, as well as the relationship between genetically-determined serum SLPI with HF, in a multi-ethnic cohort.

## Results

### Participant characteristics

Baseline characteristics of the 2,297 participants in the final analytic cohort, stratified by quartile of serum SLPI, are presented in Table 1. Within the analytic cohort, median serum SLPI was 44,785 pg/mL (interquartile range: 38,671–52,340 pg/mL). Participants within the highest quartile of serum SLPI were older and more likely men, African American and Hispanic, and former smokers compared to participants in lower SLPI quartiles. Additionally, participants with higher serum SLPI had higher systolic and diastolic blood pressure, higher low-density lipoprotein cholesterol, higher triglycerides, higher fasting glucose, higher C-reactive protein (CRP) levels, higher N-terminal-pro brain natriuretic peptide (NT-proBNP), higher high sensitivity troponin T, and lower levels of estimated glomerular filtration rate (eGFR). Multi-Ethnic Study of Atherosclerosis (MESA) participants with missing serum SLPI levels or pre-existing cardiovascular disease (CVD) were excluded. Compared with participants in the final analytic cohort, MESA participants who were excluded from this analysis were older, had higher prevalence of hypertension, and more likely self-identified as White at baseline examination. In addition, excluded participants had lower eGFR, higher systolic blood pressure, and higher CRP at baseline compared to participants included in this analysis (Supplemental Fig. S1, Supplemental Table S1).

**Table 1:** Participant characteristics at baseline (examination 2) by quartile of serum SLPI.

	Quartile 1 (n = 577)	Quartile 2 (n = 572)	Quartile 3 (n = 574)	Quartile 4 (n = 574)	P-value
Age, years, mean ± SD	59.5 ± 9.3	61.9 ± 9.7	64.3 ± 9.5	66.3 ± 9.9	< 0.001
Race/ethnicity					< 0.001
Black, n (%)	126 (21.8)	135 (23.6)	130 (22.6)	162 (28.2)	
White, n (%)	149 (25.8)	140 (24.5)	163 (28.4)	117 (20.4)	
Hispanic, n (%)	121 (21.0)	143 (25.0	143 (24.9)	175 (30.5)	
Chinese, n (%)	181 (31.4)	154 (26.9)	138 (24.0)	120 (20.9)	
Men, n (%)	205 (35.5)	258 (45.1)	301 (52.4)	316 (55.1)	< 0.001
Systolic blood pressure, mmHg, mean ± SD	118.6 ± 18.6	123.0 ± 20.1	126.5 ± 21.6	128.1 ± 21.1	< 0.001
Diastolic blood pressure, mmHg, mean ± SD	69.0 ± 9.7	70.2 ± 9.5	71.3 ± 10.1	71.5 ± 10.1	< 0.001
Anti-hypertensive medication n (%)	183 (31.7)	199 (34.8)	243 (42.3)	298 (51.9)	< 0.001
Body mass index, kg/m^2^, mean ± SD	27.8 ± 5.9	27.9 ± 5.7	27.8 ± 5.2	28.2 ± 5.2	0.57
Diabetes mellitus					< 0.001
Normal glucose, n (%)	404 (70.0)	403 (70.5)	394 (68.6)	346 (60.3)	
Impaired fasting glucose, n (%)	81 (14.0)	103 (18.0)	93 (16.2)	109 (19.0)	
Untreated diabetes, n (%)	13 (2.3)	11 (1.9)	21 (3.7)	19 (3.3)	
Treated diabetes, n (%)	79 (13.7)	55 (9.6)	66 (11.5)	100 (17.4)	
eGFR, mL/min/1.73m^2^, mean ± SD	86.6 ± 13.9	80.1 ± 14.0	76.6 ± 14.4	71.4 ± 17.4	< 0.001
CRP, mg/L, median [IQR]	1.4 [0.6, 3.5]	1.5 [0.6, 3.9]	1.7 [0.7, 3.7]	2.2 [1.0, 4.5]	< 0.001
Total cholesterol, mg/dL, mean ± SD	189.0 ± 31.8	192.2 ± 34.4	192.9 ± 36.0	193.8 ± 37.9	0.10
LDL cholesterol, mg/dL, mean ± SD	110.8 ± 28.8	114.8 ± 32.1	115.2 ± 32.1	115.6 ± 32.8	0.04
Smoking status					< 0.001
Never, n (%)	329 (57.0)	324 (56.6)	279 (48.6)	235 (40.9)	
Former, n (%)	199 (34.5)	203 (35.5)	225 (39.2)	232 (40.4)	
Current, n (%)	49 (8.5)	45 (7.9)	70 (12.2)	107 (18.6)	
Triglycerides, mg/dL, median [IQR]	102.0 [72.0, 144.0]	111.5 [82.0, 156.0]	118.5 [81.0, 170.8]	134.0 [96.0, 179.0]	< 0.001
Glucose, mg/dL, median [IQR]	91.0 [85.0, 101.0]	91.0 [86.0, 101.0]	93.0 [87.0, 101.0]	95.0 [88.3, 107.0]	< 0.001
Urine albumin:creatinine, median [IQR]	5.3 [3.2, 9.2]	5.2 [3.4, 10.2]	5.7 [3.5, 11.6]	7.0 [3.7, 18.7]	< 0.001
Heart rate, mean ± SD	65.4 ± 8.8	65.0 ± 10.0	65.5 ± 10.0	65.9 ± 10.7	0.58
NT-proBNP, median [IQR]	36.9 [17.2, 57.4]	36.8 [17.4, 66.4]	54.8 [25.9, 89.4]	47.0 [26.3, 94.9]	0.007
High-sensitivity troponin T, median [IQR]	3.0 [3.0, 4.8]	4.0 [3.0, 6.2]	4.7 [3.0, 7.2]	5.7 [3.3, 9.3]	< 0.001
SLPI, pg/mL, median [IQR]	35,438 [33,031, 37,151]	41,737 [40,247, 43,213]	48,225 [46,411, 50,159]	59,062 [54,870, 65,499]	< 0.001

*eGFR* estimated glomerular filtration rate, *HDL* high-density lipoprotein, *LDL* low-density lipoprotein, *NT-proBNP* N-terminal-pro brain natriuretic peptide, *SLPI* secretory leukocyte protease inhibitor.

### Association of serum SLPI levels with incident heart failure

**Figure 1:**
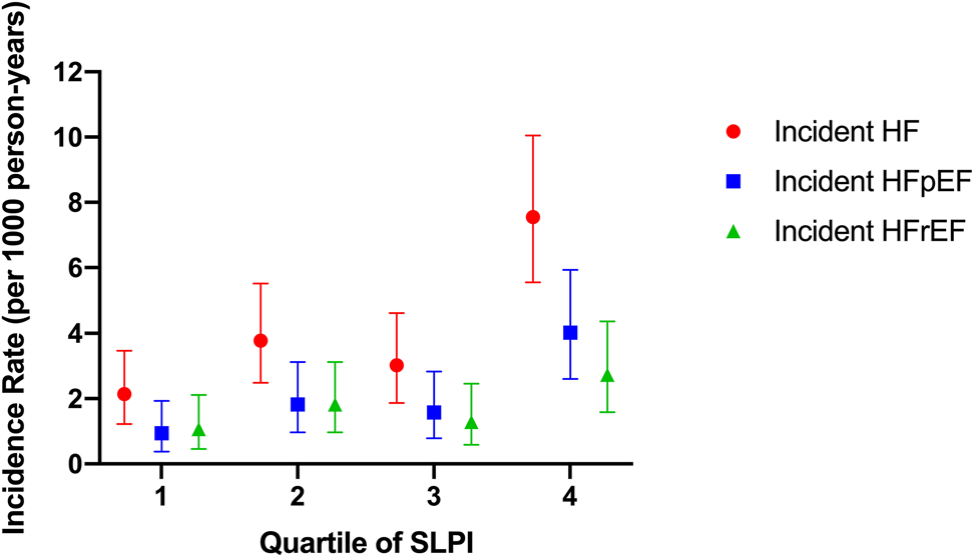
Incidence rates for heart failure (including subtypes) by quartile of serum SLPI. Unadjusted incidence rates for total heart failure (HF), HF with preserved ejection fraction (HFpEF), and HF with reduced ejection fraction (HFrEF). Error bars represent 95% confidence interval (CI).

Over a median follow-up of 13.9 years (interquartile range, 11.4–14.6 years), there were 111 total HF events. The incidence rate (per 1000 person-years) for HF was highest among participants in the highest quartile of serum SLPI (Fig. 1). On spline analysis, higher serum SLPI was linearly associated with greater risk of HF (non-linearity *P* = 0.68). (Supplemental Fig. S2). After adjustment for all chosen covariates, each doubling of serum SLPI was independently associated with 77% increased risk of HF (HR 1.77; 95% CI 1.02–3.02; *P* = 0.04) (Table 2). Among 350 participants of the analytic cohort with available NT-proBNP levels at Exam 1 or 2, there was a strong association between serum SLPI and NT-proBNP levels (β coefficient per doubling of SLPI = 0.85, standard error = 0.24, *P* ≤ 0.001) (Supplemental Fig. S3).

**Table 2:** Associations of serum SLPI with incident heart failure (including subtypes).

Outcome	Number of events/participants	Model 1		Model 2		Model 3	
		HR per doubling of SLPI (95th CI)	P-value	HR per doubling of SLPI (95th CI)	P-value	HR per doubling of SLPI (95th CI)	P-value
Any heart failure	111/2297	1.91 (1.17–3.10)	0.01	1.78 (1.04–3.04)	0.04	1.77 (1.02–3.02)	0.04
Heart failure with preserved ejection fraction	56/2297	2.27 (1.21–4.25)	0.01	2.44 (1.23–4.83)	0.01	2.44 (1.23–4.84)	0.01
Heart failure with reduced ejection fraction	47/2297	1.21 (0.52–2.85)	0.65	0.97 (0.37–2.51)	0.95	0.95 (0.36–2.46)	0.91

Hazard ratios (HR) for heart failure (including subtypes) for each model. The HR is presented as per doubling of SLPI and interpreted as per one unit on the log base 2 scale of SLPI measurement with 95% confidence intervals (CI). Model 1 adjusted for age, race, and gender. Model 2 further adjusted for body mass index (BMI), systolic blood pressure, antihypertensive medication treatment, diabetes mellitus, smoking, total cholesterol, and estimated glomerular filtration rate (eGFR). Model 3 additionally adjusted for C-reactive protein (CRP).

### Association of serum SLPI levels with incident heart failure subtypes

**Figure 2:**
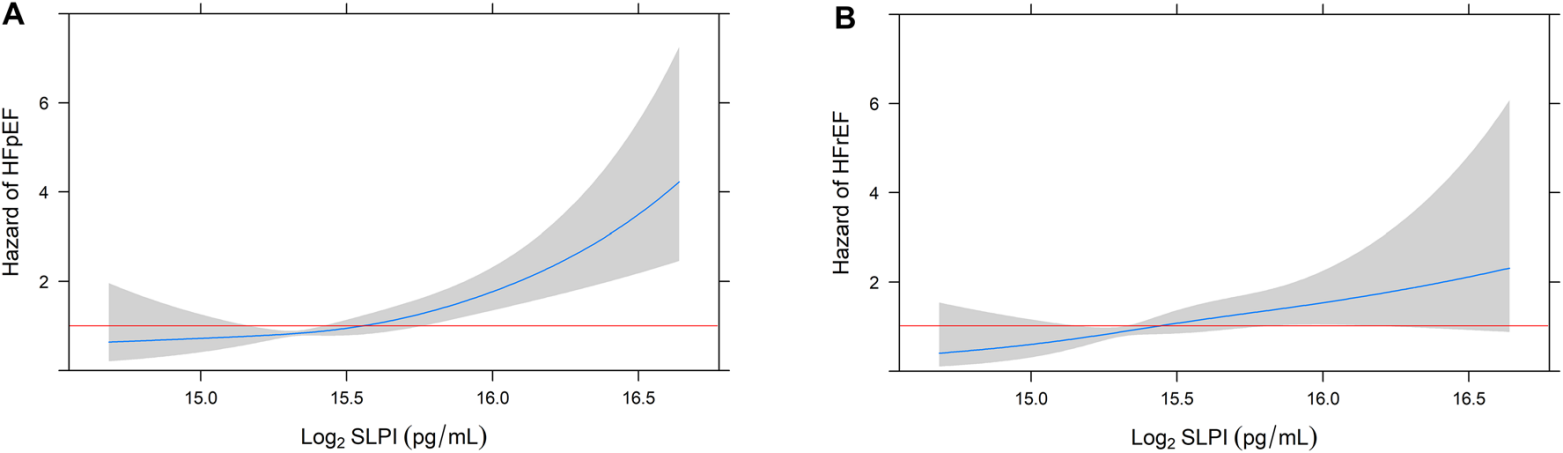
Association of serum SLPI with heart failure subtypes. (**A**) Restricted cubic spline curves of the continuous association of SLPI with heart failure with preserved ejection fraction (HFpEF). (**B**) Restricted cubic spline curves of the continuous association of SLPI with heart failure with reduced ejection fraction (HFrEF). Log base 2 transformation of SLPI can be interpreted as “per doubling.” Horizontal red line indicates a hazard ratio (HR) of 1. Shaded areas represent 95% confidence interval (CI).

Of the 111 HF events, 56 were incident hospitalization for HF with preserved ejection fraction (HFpEF), 47 were incident hospitalization for HF with reduced ejection fraction (HFrEF), and 8 were incident hospitalization for HF of unknown ejection fraction (EF). On evaluation of incident HF by EF categories, the incidence rates for both HFpEF and HFrEF were highest among individuals at the highest quartile of serum SLPI. (Fig. 1). On spline analysis, higher serum SLPI was associated with progressively greater risk of HFpEF; the effect size for risk of HFrEF appeared lower than that for HFpEF (Fig. 2).

**Figure 3:**
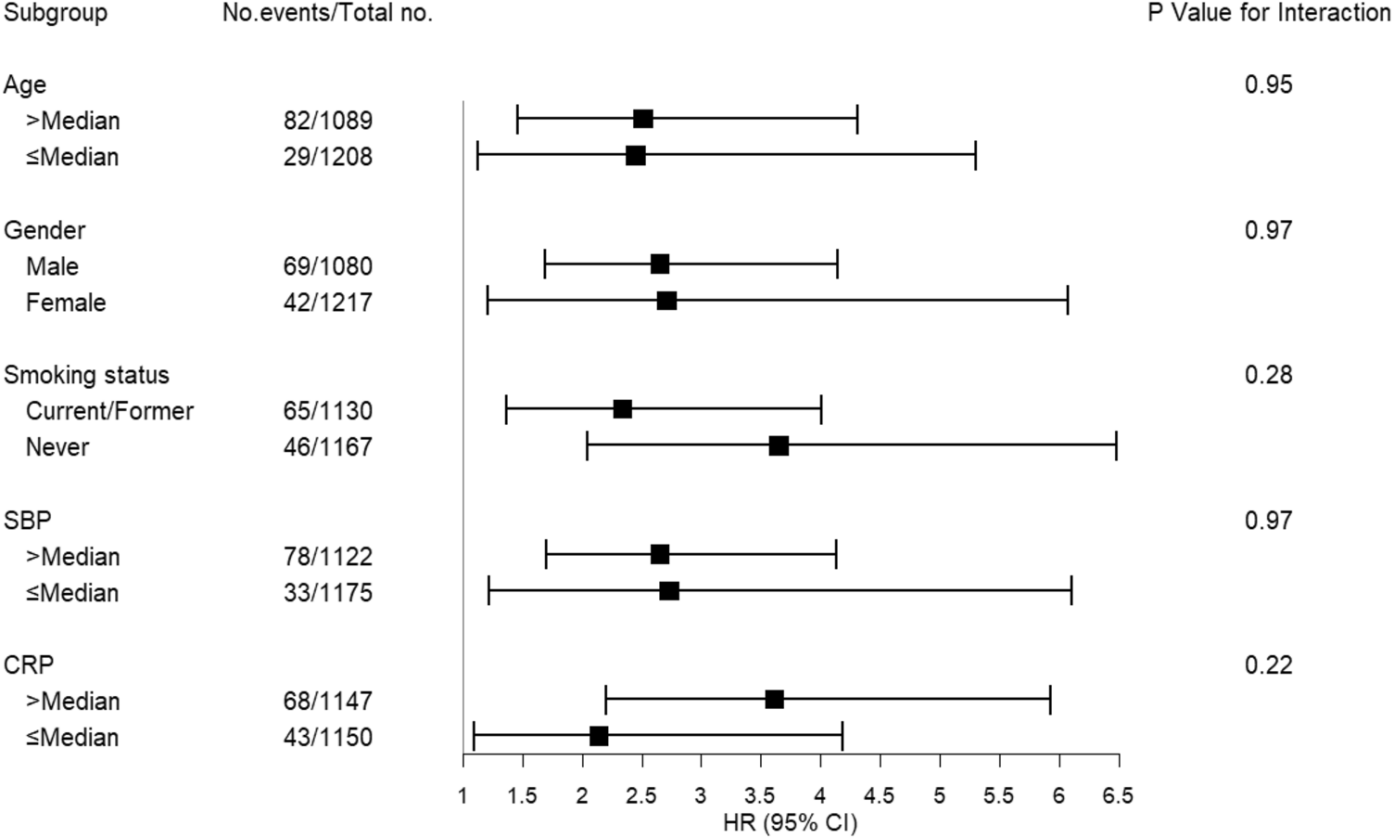
Association of serum SLPI and incident heart failure among subgroups. Cox proportional hazards models incorporating prespecified interaction terms were used to evaluate the associations of SLPI with incident heart failure in certain subgroups, including age, gender, smoking status, systolic blood pressure (SBP), and C-reactive protein (CRP). Error bars represent 95% confidence interval (CI) of hazard ratio (HR).

The associations of serum SLPI with risk of incident HFpEF and HFrEF are shown in Table 2. Associations of SLPI with incident HF with unknown EF are not shown due to limited number of such events. In unadjusted analyses, higher serum SLPI at baseline was associated with increased risk of incident HFpEF hospitalization, but not HFrEF. After adjustment for all chosen covariates, each doubling of SLPI was significantly associated with incident HFpEF (HR 2.44; 95% CI 1.23–4.84; *P* = 0.01), with higher overall magnitude of risk as all HF. Serum SLPI was not associated with risk of HFrEF (HR 0.95; 95% CI 0.36–2.46; *P* = 0.91) in fully adjusted models. While the association between serum SLPI and incident all HF was attenuated after additional adjustment for high-sensitivity troponin T in sensitivity analysis, the association between SLPI and incident HFpEF remained significant after adjustment for high-sensitivity troponin-T (HR 2.23; 95% CI 1.13–4.24; *P* = 0.02) (Supplemental Table S2). Upon subgroup analyses, the associations of serum SLPI with incident HF were consistent across pre-specified age, gender, smoking, systolic blood pressure, and CRP subgroups (Fig. 3).

### Association of previously reported circulating SLPI pQTLs with HF events in MESA

Two recent GWAS of the human blood plasma proteome in European ancestry cohorts identified multiple SNPs associated with circulating plasma SLPI protein levels^12,13^. We examined the relationships of these previously identified SLPI pQTLs with HF events in MESA. In total, we assessed three previously identified pQTLs that were either directly genotyped (rs3863292) or imputed (rs16920858, rs7205804) in MESA. There was no significant association between any of the three previously identified SLPI pQTLs with HF events in MESA participants after Bonferroni correction (Supplemental Table S3). In ancestry-specific and trans-ancestral analyses of MESA participants, we were unable to replicate the previously reported association of these variants with serum SLPI levels in MESA participants (Supplemental Table S4).

## Discussion

**Figure 4:**
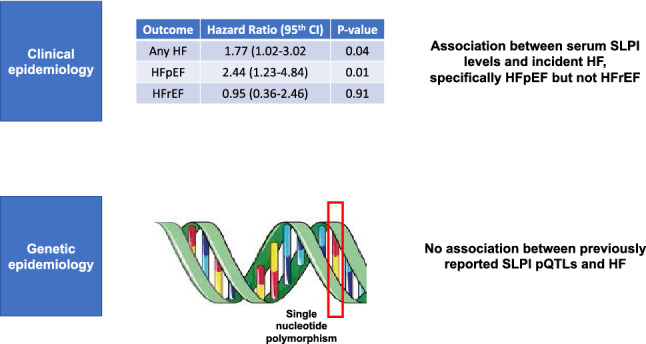
Central Illustration. See text for details. *CI* confidence interval, *HF* heart failure, *HFpEF* heart failure with preserved ejection fraction, *HFrEF* heart failure with reduced ejection fraction, *pQTLs* protein quantitative trait loci, *SLPI* secretory leukocyte protease inhibitor.

The role of serum SLPI in incident HF is not well understood. In this multi-ethnic cohort of adults without CVD at baseline, we find substantial variation in serum SLPI levels. Participants with higher serum SLPI levels had higher systolic and diastolic blood pressure, higher low-density lipoprotein cholesterol, higher triglycerides, higher fasting glucose, higher CRP, higher NT-proBNP, and lower levels of eGFR. After adjustment for demographic and cardiovascular risk factors, baseline serum SLPI levels were significantly associated with higher risk for incident HF. On evaluation of HF subtypes, serum SLPI was strongly associated with incident HFpEF, but not HFrEF. The association of serum SLPI with incident HF was consistent across pre-specified subgroups, including inflammation as measured by CRP. Previously reported SLPI pQTLs were not associated with HF events in MESA participants, which may be due to a smaller sample size and small effect sizes of these pQTLs. Taken together, these data are consistent with the hypothesis that circulating SLPI levels may be an inflammatory correlate that sheds light upon the pathobiology of HFpEF (Fig. 4).

SLPI is not traditionally described as a systemic antiprotease. In contrast to circulating systemic antiproteases such as α_1_-antitrypsin and α2-macroglobulin, SLPI has classically been considered a type of “alarm antiprotease” that is produced locally in response to inflammatory cytokines and bacterial products with limited systemic expression^16,17^. This study suggests that significant levels of SLPI are found in the systemic circulation with sufficient variation to be used as a biomarker of disease.

We observed that participants within the highest quartile of serum SLPI were more likely men, African American and Hispanic, and former smokers compared to participants in lower SLPI quartiles. The higher levels of serum SLPI among men compared to women may be related to sex-specific differences in inflammation already present in mid-adulthood. Sex hormones have also been implicated in the regulation of SLPI expression^18^. The higher levels of serum SLPI among self-reported African American and Hispanics is less likely to be due to ancestral genetic variation given that previously reported SLPI pQTLs were not associated with serum SLPI levels in MESA participants in trans-ancestral or ancestry-specific analyses. Smoking is a significant risk factor for CVD and is known to transcriptionally increase SLPI expression through signal transducers and activators of transcription 1 (STAT1)^19^. However, the observed relationship between serum SLPI and HF was robust and independent of these potential confounders. Additionally, the associations of serum SLPI with incident HF were consistent across pre-specified age, gender, and smoking subgroups.

We demonstrate for the first time the prospective associations of baseline serum SLPI with symptomatic HF, as defined by incident HF hospitalization. The association of SLPI with HF was independent of systemic inflammation as measured by CRP levels. This suggests that SLPI may reflect cardiovascular inflammatory pathways distinct from those captured by CRP. This may be due to the different physiological responses to inflammation between CRP and SLPI^20^. While CRP is a hepatically-derived acute phase reactant that increases primarily in response to interleukin (IL)-6 secretion by macrophages and T cells, *SLPI* is transcriptionally upregulated in myeloid cells by toll-like receptor ligands and pattern recognition receptor ligands^21^. Additionally, *SLPI* mRNA expression in macrophages is upregulated by multiple interleukins, including IL-6 and IL-10^22^. Additional studies are needed to identify the specific inflammatory pathways that differentially upregulate CRP and SLPI in HF.

Interestingly, the association between SLPI and HF was driven by incident HFpEF but not HFrEF upon subgroup analysis. This differential association is notable because while many circulating proteins are associated with incident HFrEF, there are very few specific for incident HFpEF^23–28^. The SLPI-HFpEF association is also in accordance with the different contributions of inflammation to the pathophysiology of HFpEF as compared with HFrEF^29^. HFpEF is a systemic, multi-organ disorder, and previous work suggests that HFpEF may be the result of a comorbidity-induced systemic low-grade inflammatory state^30^. Consistent with this paradigm, we found that serum SLPI levels are positively correlated with multiple HFpEF comorbidities, including hypertension, diabetes, renal insufficiency, smoking, and dyslipidemia. Therefore, SLPI may partially reflect comorbidity-induced systemic inflammation in HFpEF. Meanwhile, the inflammation in HFrEF is hypothesized to be predominantly the result of direct myocardial injury and cardiomyocyte loss, which may not be sufficient to raise systemic SLPI levels. This is consistent with the GTEx database which demonstrates that SLPI is minimally expressed in the human left ventricle or atrial appendage. Additionally, on sensitivity analysis adjusting for high-sensitivity troponin T, only the association between SLPI and incident HFpEF remained consistent. In aggregate, our findings raise the possibility that SLPI-mediated anti-inflammatory pathways could be of particular therapeutic importance for HFpEF, a HF subtype that is in need of additional effective interventions.

Our clinical findings are also consistent with previous in vitro and in vivo animal models suggesting that SLPI is important for cardiac function. Overexpression of human SLPI in immortalized cardiomyocytes reduced cell death and injury in an in vitro model of ischemia/reperfusion injury^31^. The administration of recombinant SLPI also improved early recovery of cardiac function in a mouse model of cardiac transplantation and was associated with reduced protease activity and transforming growth factor (TGF)-β expression^32^. In addition to its secreted form, SLPI also has an intracellular form which has not been studied in cardiovascular models and may exert unique functions^33^.

Important insights into the mechanistic role of SLPI in HF may be found in prior studies of SLPI in different organ systems. In chronic obstructive pulmonary disease (COPD) and cystic fibrosis, SLPI protects against lung injury from excessive inflammatory immune responses. However, despite higher levels of SLPI in the sputum of patients with COPD, this upregulation of SLPI is ultimately insufficient to counteract the massive overproduction of proteases in advanced lung disease^34^. Meanwhile, inhibition of SLPI using a blocking antibody was associated with neutrophil accumulation and significantly increased lung injury^35^. It is possible that a similar mechanism exists in HFpEF, such that systemic SLPI expression is induced by low-level chronic inflammation, but ultimately the cardioprotective effects of endogenous SLPI production are overwhelmed by excessive inflammatory signaling in progressive HFpEF. Thus, we hypothesize that serum SLPI not only serves as a risk marker, but may also be cardioprotective, similar to B-type natriuretic peptide (BNP)^32^. Future studies with larger cohorts using multi-biomarker panels are also needed to definitively determine the potential role of serum SLPI as a biomarker of HFpEF risk using risk prediction modeling.

Previously reported SLPI pQTLs among Europeans were not associated with HF events in MESA participants^12,13^. We were also unable to replicate previously reported SLPI pQTLs among Europeans with serum SLPI levels in MESA participants. This lack of association may be explained by differences in sample size, genetic ancestry, environmental factors, or assay measurement between the previously studied European populations and participants in MESA. As such, larger studies with genetic data, serum SLPI levels, and a greater number of HF events are required to further elucidate whether genetically-mediated SLPI levels are associated with HF. These findings also underscore the importance of validating previously identified pQTLs with circulating protein levels across different population-based studies, particularly among populations of different ancestries. Given that we observed higher serum SLPI levels in participants with hypertension, hyperlipidemia, hyperglycemia, and kidney dysfunction, future analyses may also consider gene-environment interactions or epigenetic regulation of serum SLPI levels as a result of these acquired comorbidities.

Our study has several strengths. We used a combination of epidemiologic and genomic data from a large, ethnically diverse, community-based cohort that has been well characterized through longitudinal in-person examinations with a median follow up of 13.9 years to determine the development of HF. Incident HF was rigorously defined as hospitalization with evidence for imaging, treatment, or physician diagnosis of HF within the medical record, thus this definition was highly specific for symptomatic HF. Our study was the first to examine the association of SLPI with HF (including subtypes), which held true after adjusting for traditional inflammatory risk as measured by CRP. This is also the first study, to our knowledge, assessing SLPI pQTLs with incident HF in a multi-ethnic cohort.

The results of this study should be interpreted in the context of certain limitations. Although our final analytic cohort was large, several participants were excluded because of lack of serum SLPI measurement or pre-existing CVD. These excluded participants represented a slightly higher-risk group with a higher prevalence of cardiovascular risk factors, and thus our final analytic cohort may underestimate associations. Additionally, while we adjusted for various demographic, clinical, and laboratory covariates, our findings remain subject to potential residual confounding. Furthermore, incident HF hospitalizations (and their subtypes) were relatively low in this cohort, and the requirement of hospitalization for HF adjudication prevents detection of mild, outpatient cases of HF. Population-based external validation cohorts with high HF event rates are required to verify our findings of specific associations of serum SLPI with these incident CVD outcomes. While we did not adjust for NT-proBNP in our models assessing the association between SLPI and incident HF given the high degree of missingness of NT-proBNP at Exam 1 or 2 (n = 1947), elevated natriuretic peptides may mediate the association between SLPI-related inflammation and HF given the strong positive relationship between SLPI and NT-proBNP in our study. Additional investigations are also required to understand drivers of circulating SLPI expression, the role of cumulative SLPI exposure in HF risk, and clinically meaningful changes in SLPI over time.

In summary, baseline serum SLPI was significantly associated with HF over long-term follow-up in this multi-ethnic, community-based cohort. Notably, the increased risk of HF associated with SLPI was driven by incident HFpEF, but not HFrEF. The association of SLPI with HF was consistent across pre-specified subgroups, including baseline CRP level, suggesting that SLPI may reflect cardiovascular inflammatory pathways distinct from those captured by CRP. We did not observe a significant association between previously reported SLPI pQTLs and HF in MESA participants. Additionally, there was no significant association between previously reported SLPI pQTLs and serum SLPI levels in MESA participants, demonstrating importance of validating previously identified pQTLs with circulating protein levels across different population-based studies, particularly with diverse individuals. Additional molecular and larger, multi-ethnic population genomic studies are required to fully understand the potential mechanisms behind SLPI and incident HFpEF.

## Methods

### Study participants

The Multi-Ethnic Study of Atherosclerosis (MESA) study, as previously described, is a prospective cohort of 6,814 community-dwelling adults aged 45–84 years designed to understand the risk factors, prevalence, and progression of subclinical cardiovascular disease (CVD)^36^. In brief, participants who identified themselves as Black, Chinese, Hispanic, or non-Hispanic White were recruited between 2000 and 2002 across 6 study sites in the United States (Baltimore, MD; Chicago, IL; St. Paul, MN; Forsyth County, NC; New York, NY; and Los Angeles, CA). At the time of recruitment, participants had no history of CVD, defined as myocardial infarction, angina, stroke, transient ischemic attack, heart failure (HF), atrial fibrillation, nitroglycerin use, angioplasty, pacemaker or defibrillator, or cardiac surgery. After recruitment and a baseline in-person examination (examination 1), 5 additional follow-up in-person examinations were completed at 2–5 year intervals. Examinations included standardized questionnaires that collected information on demographics, medical history, and medication use. Resting blood pressure and blood sampling were also obtained during examinations. Diabetes mellitus was defined as self-reported diagnosis, fasting glucose ≥ 126 mg/dL, or use of antidiabetic medication. Estimated glomerular filtration rate (eGFR) was calculated by the Chronic Kidney Disease Epidemiology Collaboration equation using examination 1 serum creatinine. Baseline C-reactive protein (CRP) levels were collected at examination 1. Baseline N-terminal-pro brain natriuretic peptide (NT-proBNP) levels were collected at examination 1 or 2. Baseline high-sensitivity troponin T (hs-troponin T) levels were collected at examination 1 or 2.

For this analysis, we included participants with available serum secretory leukocyte protease inhibitor (SLPI) levels at examination 2 (conducted between 2002 and 2004) and available data on baseline covariates and follow-up. SLPI levels were measured as part of the MESA Adhesion Ancillary Study^37^. Of the 6814 individuals in MESA, 4517 were excluded: 4373 did not have SLPI levels drawn, 11 had CVD prior to Exam 2 (either HF or coronary heart disease), and 133 were missing covariate data at Exam 2. (Supplemental Fig. S1). Thus, the final analytic cohort for this analysis was 2297 participants. The MESA study protocol and its ancillary studies was approved by the institutional review board of each study site; all participants provided informed consent. All methods were performed in accordance with the relevant guidelines and regulations. Additional data that support the findings of this study are available from the corresponding author upon reasonable request.

### SLPI measurement

At examination 2 (2002–2004), blood samples were obtained from participants after overnight fasting and were stored at − 70 °C. As part of the MESA Adhesion Ancillary Study, serum SLPI was measured by quantitative sandwich enzyme-linked immunosorbent assay (ELISA; R&D Systems, Minneapolis, MN)^37^. The interassay coefficient of variation was 8.9% at a mean concentration of 36,888 pg/mL and the minimum detectable level was 25 pg/mL.

### Heart failure assessment

Incident hospitalized HF was adjudicated by 2 study physicians blinded to other study data through previously described medical record review^36^. In brief, the participants of MESA were screened for clinical events through regular telephone contact and in‐person examinations. All identified records from hospitalizations for CVD events were abstracted, and MESA personnel transmitted records of symptoms, medical history, biomarkers, electrocardiograms, echocardiograms, cardiac catheterization reports, other imaging studies, and outpatient records (if available) to the MESA coordinating center. HF events were defined as definite or probable. Both definite and probable HF events required symptoms of HF, including shortness of breath or edema. Definite HF was additionally defined on the basis of ≥ 1 of the following: pulmonary edema on chest radiography, left ventricular dilation or decreased systolic function, or evidence of diastolic dysfunction. If criteria for definite HF were not available, probable HF was defined as a physician diagnosis of HF in the clinical record and documentation of medical treatment for HF. The primary outcome of interest in this analysis was incident hospitalized HF, defined as any probable or definite HF event. We additionally evaluated HF subtypes (HF with preserved ejection fraction [HFpEF], HF with reduced ejection fraction [HFrEF], and HF with unknown ejection fraction) as secondary outcomes. HFpEF was defined as a HF event with documentation of left ventricular ejection fraction (EF) ≥ 45% on echocardiogram or radionucleotide study at time of hospitalization, and HFrEF was defined as EF < 45% at hospitalization. HF with unknown EF was defined as a HF event without documented EF on imaging study at time of hospitalization. Event ascertainment of HF in MESA has been fully updated and completed through 2017.

### Genotyping and imputation

MESA participants were genotyped using the Affymetrix Genome-Wide Human SNP Array 6.0 through the MESA Candidate Gene Association Resource (CARe) and MESA SHARe projects. Of the 2297 participants in the final analytic cohort, genomic data was available for 2231 participants. The participants were stratified by self-reported race/ethnicity. Genotype imputation was performed using IMPUTE (version 2.1.0)^38^ and HapMap Phase I and II reference panels (release #22, National Center for Biotechnology Information Build 36 [dbSNP b126]). Black, Chinese, and Hispanic participants were imputed using the CEU + YRI + CHB + JPT reference panels and non-Hispanic White participants were imputed using the CEU reference panels.

### Analyses of previously reported SLPI protein quantitative trait loci (pQTL)

A previous GWAS conducted in the Cooperative Health Research in the Region of Augsburg (KORA) Study investigating associations between SNPs and 1,124 plasma protein levels identified four SNPs associated with circulating SLPI^12^. Of these four SNPs, one was directly genotyped (rs3863292) and one was imputed (rs16920858) in MESA. A previous GWAS conducted in 35,559 Icelanders investigating associations between SNPs and 4719 plasma proteins identified an additional seven SNPs associated with circulating SLPI^13^. While none of these seven SNPs were directly genotyped in MESA, one SNP was imputed (rs7205804). We evaluated the associations of these three previously identified SNPs with HF events in trans-ancestral analyses in MESA participants. We also evaluated the associations of these three previously identified SNPs with serum SLPI levels in ancestry-specific and trans-ancestral analyses in MESA participants. A Bonferroni-corrected *P* < 0.0166 was considered statistically significant.

### Statistical analysis

Demographic and clinical characteristics at baseline examination 2 (2002–2004) were compared by quartile of SLPI using χ^2^ tests for categorical variables and univariate general linear models for continuous variables. Multivariable Cox proportional hazards regression models were used to evaluate the associations of SLPI with incident HF and its subtypes (HFpEF and HFrEF). We evaluated SLPI as a continuous variable after log base 2 transformation, which can be interpreted as “per doubling.” Exam 2, the time of SLPI measurement, was defined as the time origin for this analysis. The proportionality of hazards assumption was confirmed by Schoenfeld goodness-of-fit procedures. We first assessed the potential nonlinear associations of SLPI and hazard of incident HF using separate restricted cubic splines with 3 knots in Cox proportional hazards regression. We assessed the associations of SLPI as a continuous variable (per doubling) with incident HF and its subtypes. We evaluated the time to first HF subtype event using separate Cox proportion hazards models with censoring on the opposing type. The hazard ratio is interpreted as per one unit on the log base 2 scale of SLPI measurement. For all Cox regression models, covariates were obtained at examination 2, except for eGFR and CRP, which were obtained at examination 1. Model 1 adjusted for age, race, and sex. Model 2 further adjusted for body mass index (BMI), systolic blood pressure, antihypertensive medication treatment, diabetes mellitus, smoking, total cholesterol, and eGFR. Model 3 additionally adjusted for CRP. In sensitivity analysis, we further adjusted for hs-troponin T in addition to Model 3 covariates. We assessed for effect modification of age, sex, smoking status, systolic blood pressure, and CRP on the association of SLPI with incident HF using interaction terms for age (above vs. below median), sex, smoking status (current/former vs. never), systolic blood pressure (above vs. below median), and CRP (above vs. below median). A two‐tailed *P* < 0.05 was considered statistically significant. Statistical analysis was performed using R version 4.0.2 (Vienna, Austria).

## Supplementary Information

Supplementary Information.

## Data Availability

The genomic dataset analyzed during the current study are publicly available in the dbGAP repository (Study Accession: phs000209.v13.p3). The ELISA dataset analyzed during the current study are publicly available in the NHLBI’s Biologic Specimen and Data Repository Information Coordinating Center repository (https://biolincc.nhlbi.nih.gov/studies/mesa).

## Abbreviations

CRP: C-reactive protein
CVD: Cardiovascular disease
HF: Heart failure
HFpEF: Heart failure with preserved ejection fraction
HFrEF: Heart failure with reduced ejection fraction
MESA: Multi-Ethnic Study of Atherosclerosis
pQTL: Protein quantitative trait loci
SLPI: Secretory leukocyte protease inhibitor
SNP: Single nucleotide polymorphism
