# Supplementary material for: Secretory leukocyte protease inhibitor and risk of heart failure in the Multi-Ethnic Study of Atherosclerosis
Source: Sci Rep. 2023 Jan 12;13:604. doi: 10.1038/s41598-023-27679-0 (PMC9837113; doi:10.1038/s41598-023-27679-0)
Supplement: Supplementary file 1 — Supplementary Information. [file 41598_2023_27679_MOESM1_ESM.docx]

**SUPPLEMENTAL MATERIAL**

**Figure S1. Flow diagram for study inclusion.**

SLPI = secretory leukocyte protease inhibitor; CHD = coronary heart disease; HF = heart failure.

**Figure S2. Association of serum SLPI with heart failure.**

2Restricted cubic spline curves of the continuous association of SLPI with incident heart failure (HF). Log base 2 transformation of SLPI can be interpreted as “per doubling.” Horizontal red line indicates a hazard ratio (HR) of 1. Shaded areas represent 95% confidence interval (CI).

**Figure S3. Association of serum SLPI with NT-proBNP at Exam 1 or 2.**


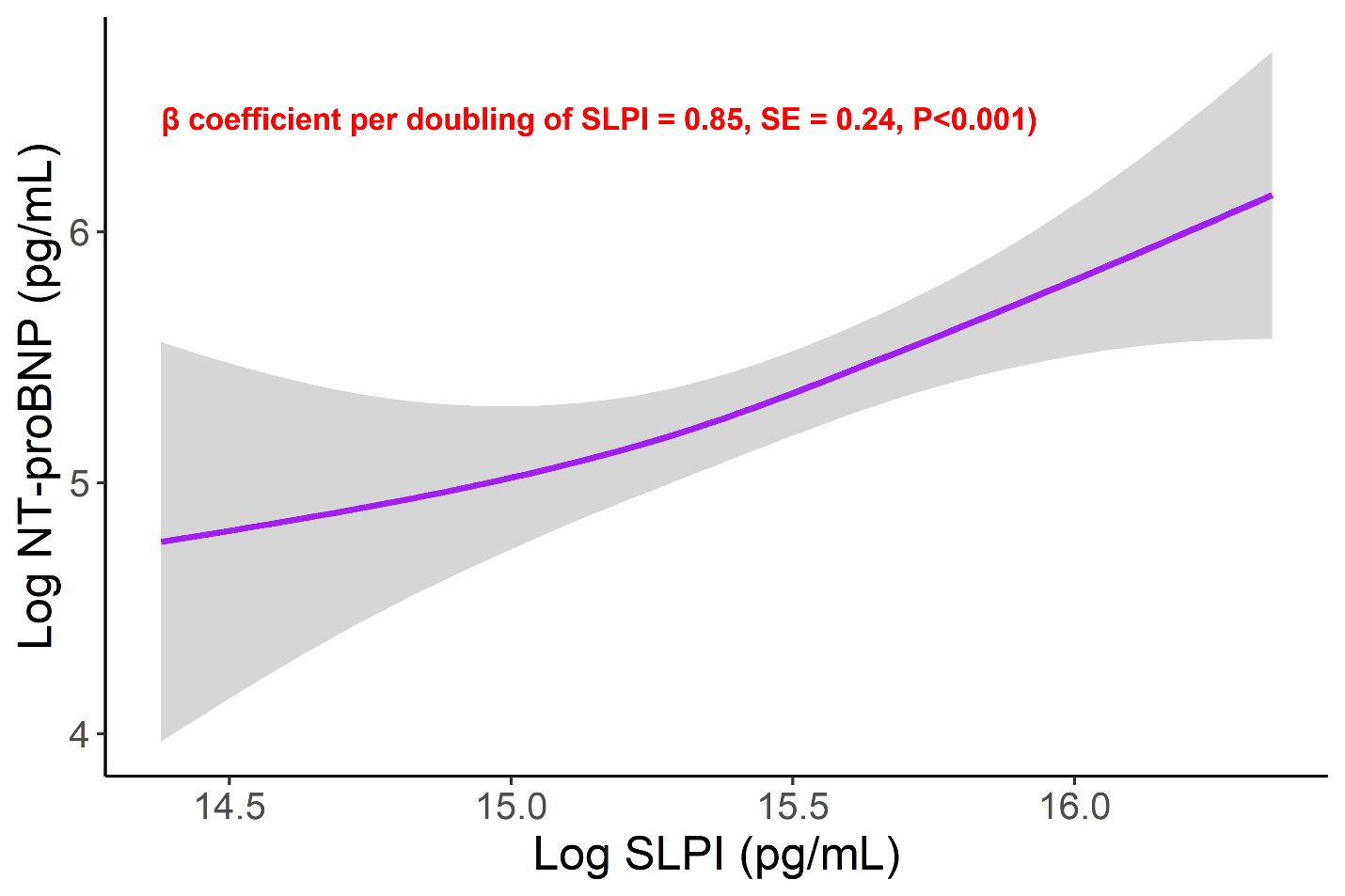


Log base 2 transformation can be interpreted as “per doubling.” Shaded areas represent 95% confidence interval (CI). SLPI = secretory leukocyte protease inhibitor; NT-proBNP = N-terminal-pro brain natriuretic peptide.

**Table S1. Clinical characteristics at Exam 2 by study inclusion status.**

|  | Excluded  (n=4516) | Included  (n=2297) | P value |
| --- | --- | --- | --- |
| Age, y, mean ± SD | 64.0 ± 10.2 | 63.0 ± 9.9 | <0.001 |
| Men, n (%) | 1889 (48) | 1080 (47) | 0.468 |
| Race/Ethnicity |  |  | <0.001 |
| Black, n (%) | 1136 (29) | 553 (24) |  |
| White, n (%) | 1895 (48) | 569 (25) |  |
| Hispanic, n (%) | 768 (19.5) | 582 (25) |  |
| Chinese, n (%) | 136 (3.5) | 593 (26) |  |
| Body mass index, kg/m^2^, mean ± SD | 28.6 ± 5.5 | 27.9 ± 5.5 | <0.001 |
| Systolic blood pressure, mmHg, mean ± SD | 124.5 ± 20.9 | 124.1 ± 20.7 | 0.292 |
| Diastolic blood pressure, mmHg, mean ± SD | 70.4 ± 10.2 | 70.5 ± 9.8 | 0.766 |
| Anti-hypertensive medication n (%) | 1587 (43.2) | 923 (40.2) | 0.023 |
| Diabetes mellitus, n (%) | 546 (14) | 364 (15.9) | 0.266 |
| Smoking status |  |  | <0.001 |
| Current smoker, n (%) | 424 (10.9) | 271 (11.8) |  |
| Former smoker, n (%) | 1689 (43.4) | 859 (37.4) |  |
| Never smoker, n (%) | 1778 (45.7) | 1167 (50.8) |  |
| Total cholesterol, mg/dL, mean ± SD | 190.9 ± 36.2 | 192.0 ± 35.1 | 0.259 |
| LDL cholesterol, mg/dL, mean ± SD | 113.3 ± 32.5 | 114.1 ± 31.5 | 0.343 |
| Triglycerides, mg/dL, median [IQR] | 110.0 [78.0, 157.0] | 114.0 [81.0, 163.0] | 0.003 |
| eGFR, mL/min/1.73m^2^, mean ± SD | 77.3 ± 16.4 | 78.7 ± 15.9 | 0.001 |
| CRP, mg/L, median [IQR] | 2.0 [0.9, 4.4] | 1.7 [0.7, 3.9] | <0.001 |

eGFR = estimated glomerular filtration rate; LDL = low density lipoprotein; CRP = C-reactive protein; SD = standard deviation; IQR = interquartile range.

**Table S2. Associations of serum SLPI with incident heart failure (including subtypes) with additional adjustment for high-sensitivity troponin T.**

| **Outcome** | **Model** | |
| --- | --- | --- |
|  | HR per doubling of SLPI (95^th^ CI) | P-value |
| **Any heart failure** | 1.54 (0.89-2.68) | 0.13 |
| Heart failure with preserved ejection fraction | 2.24 (1.13-4.42) | 0.02 |
| Heart failure with reduced ejection fraction | 0.60 (0.21-1.68) | 0.33 |

Hazard ratios (HR) for heart failure (including subtypes). The HR is presented as per doubling of SLPI and interpreted as per one unit on the log base 2 scale of SLPI measurement with 95% confidence intervals (CI). The presented Model adjusted for all Model 3 covariates (age, race, gender, body mass index [BMI], systolic blood pressure, anti-hypertensive medication treatment, diabetes mellitus, smoking, total cholesterol, estimated glomerular filtration rate [eGFR], C-reactive protein [CRP]), and additionally adjusted for high-sensitivity troponin T collected at Exam 1 or 2.

**Table S3. Associations between previously reported SLPI pQTLs and HF events in MESA participants.**

| SNP | Chr | Position | A1/A2 | HF | |
| --- | --- | --- | --- | --- | --- |
|  |  |  |  | OR | *P* Value |
| rs16920858 | 9 | 104590175 | T/G | 1.24 | 0.451 |
| rs3863292 | 11 | 5318677 | T/C | 1.05 | 0.852 |
| rs7205804 | 16 | 57004889 | A/G | 0.64 | 0.022 |

Single nucleotide polymorphism positions are according to hg19. Chr, chromosome; A1/A2, allele 1/allele 2; HF, heart failure; OR, odds ratio.

**Table S4. Ancestry-specific and trans-ancestral association results for previously reported SLPI pQTLs with SLPI levels in MESA.**

| SNP | Chr | Position | A1/A2 | 𝛽 | *P* Value |
| --- | --- | --- | --- | --- | --- |
| Black Participants |  |  |  |  |  |
| rs16920858 | 9 | 104590175 | T/G | 0.02 | 0.560 |
| rs3863292 | 11 | 5318677 | T/C | 0.04 | 0.108 |
| rs7205804 | 16 | 57004889 | A/G | 0.02 | 0.667 |
|  |  |  |  |  |  |
| Chinese Participants |  |  |  |  |  |
| rs16920858 | 9 | 104590175 | T/G | -0.03 | 0.349 |
| rs3863292 | 11 | 5318677 | T/C | -0.10 | 0.227 |
| rs7205804 | 16 | 57004889 | A/G | 0.01 | 0.738 |
|  |  |  |  |  |  |
| Hispanic Participants |  |  |  |  |  |
| rs16920858 | 9 | 104590175 | T/G | 0.07 | 0.182 |
| rs3863292 | 11 | 5318677 | T/C | 0.03 | 0.353 |
| rs7205804 | 16 | 57004889 | A/G | -0.04 | 0.105 |
|  |  |  |  |  |  |
| White Participants |  |  |  |  |  |
| rs16920858 | 9 | 104590175 | T/G | -0.04 | 0.625 |
| rs3863292 | 11 | 5318677 | T/C | -0.04 | 0.264 |
| rs7205804 | 16 | 57004889 | A/G | 0.01 | 0.711 |
|  |  |  |  |  |  |
| Trans-Ancestral |  |  |  |  |  |
| rs16920858 | 9 | 104590175 | T/G | 0.01 | 0.855 |
| rs3863292 | 11 | 5318677 | T/C | 0.02 | 0.403 |
| rs7205804 | 16 | 57004889 | A/G | -0.01 | 0.844 |

Single nucleotide polymorphism positions are according to hg19. Chr, chromosome; A1/A2, allele 1/allele 2.
